# Effects of Providing Tailored Information About e-Cigarettes in a Web-Based Smoking Cessation Intervention: Protocol for a Randomized Controlled Trial

**DOI:** 10.2196/27088

**Published:** 2021-05-14

**Authors:** Jan Mathis Elling, Rik Crutzen, Reinskje Talhout, Hein de Vries

**Affiliations:** 1 Department of Health Promotion Maastricht University/CAPHRI Maastricht Netherlands; 2 Centre for Health Protection National Institute for Public Health and the Environment (RIVM) Bilthoven Netherlands

**Keywords:** digital health, eHealth, mHealth, electronic cigarette, e-cigarette, electronic nicotine delivery system, ENDS, smoking, smoking cessation, computer tailoring

## Abstract

**Background:**

There is an ongoing debate whether electronic cigarettes (e-cigarettes) should be advocated for smoking cessation. Because of this uncertainty, information about the use of e-cigarettes for smoking cessation is usually not provided in governmental smoking cessation communications. However, there is an information need among smokers because despite this uncertainty, e-cigarettes are used by many smokers to reduce and quit tobacco smoking.

**Objective:**

The aim of this study is to describe the protocol of a randomized controlled trial that assesses the effect of providing tailored information about e-cigarettes compared to not providing this information on determinants of decision making and smoking reduction and abstinence. This information is provided in the context of a digital smoking cessation intervention.

**Methods:**

A randomized controlled trial with a 6-month follow-up period will be conducted among adult smokers motivated to quit smoking within 5 years. Participants will be 1:1 randomized into either the intervention condition or control condition. In this trial, which is grounded on the I-Change model, participants in both conditions will receive tailored feedback on attitude, social influence, preparatory plans, self-efficacy, and coping plans. Information on 6 clusters of smoking cessation methods (face-to-face counselling, eHealth interventions, telephone counselling, group-based programs, nicotine replacement therapy, and prescription medication) will be provided in both conditions. Smokers in the intervention condition will also receive detailed tailored information on e-cigarettes, while smokers in the control condition will not receive this information. The primary outcome measure will be the number of tobacco cigarettes smoked in the past 7 days. Secondary outcome measures will include 7-day point prevalence tobacco abstinence, 7-day point prevalence e-cigarette abstinence, and determinants of decision making (ie, knowledge and attitude regarding e-cigarettes). All outcomes will be self-assessed through web-based questionnaires.

**Results:**

This project is supported by a research grant of the National Institute for Public Health and the Environment (Rijksinstituut voor Volksgezondheid en Milieu). Ethical approval was granted by the Ethics Review Committee Health, Medicine and Life Sciences at Maastricht University (FHML-REC/2019/072). Recruitment began in March 2020 and was completed by July 2020. We enrolled 492 smokers in this study. The results are expected to be published in June 2021.

**Conclusions:**

The experimental design of this study allows conclusions to be formed regarding the effects of tailored information about e-cigarettes on decision making and smoking behavior. Our findings can inform the development of future smoking cessation interventions.

**Trial Registration:**

Dutch Trial Register Trial NL8330; https://www.trialregister.nl/trial/8330

**International Registered Report Identifier (IRRID):**

DERR1-10.2196/27088

## Introduction

### Background

Tobacco smoking is a major public health threat, contributing to increased morbidity and mortality [1]. In the Netherlands, tobacco smoking is responsible for more than 19,000 deaths per year [2]. Many smokers report that they want to quit smoking, but only about 4% of smokers trying to quit without assistance succeed in their cessation attempts [3,4]. Most smokers find it hard to quit smoking because of the highly addictive nature of nicotine [5], and while they smoke for the nicotine, their probability of dying prematurely increases owing to the by-products of burnt tobacco (eg, tar) [6]. In this paper, we will describe the study protocol for a randomized controlled trial assessing an intervention aimed at quitting combustible cigarette smoking and the potential added effects of providing tailored information on electronic cigarettes (e-cigarettes).

### e-Cigarettes for Smoking Cessation

e-Cigarettes, also called as electronic nicotine delivery systems, are handheld electronic devices that generate aerosols by heating a liquid that usually contains nicotine, flavorings, and other compounds [7]. Because e-cigarettes do not burn tobacco, users are not exposed to the damaging substances of combustible tobacco [7]. However, it is important to note that, although e-cigarette aerosols generally contain fewer toxic chemicals than cigarette smoke, all tobacco (and related) products, including e-cigarettes, carry risks [8]. Smokers who want to quit smoking can use e-cigarettes as an aid for smoking reduction, cessation, and relapse prevention [7,9]. e-Cigarettes may be advantageous over nicotine replacement therapy because they are able to provide nicotine effectively and mimic the smoking experience [10]. Using e-cigarettes for smoking cessation can be considered as a tobacco harm reduction strategy [11]. There is an ongoing debate whether e-cigarettes should be advocated for smoking cessation [12]. A recent Cochrane systematic review concluded that the current evidence provides moderate certainty that e-cigarettes with nicotine are superior to e-cigarettes without nicotine and nicotine replacement therapy concerning smoking cessation [7]. Reviews on the effectiveness of using e-cigarettes for smoking cessation stress that more evidence is needed to be confident about the effects [7,8,13-15]. Furthermore, e-cigarettes developed quickly in recent years and findings from studies conducted with past generations of e-cigarettes (eg, cigalikes, battery pens) are not applicable to state-of-the-art e-cigarettes (eg, pod mods) [8]. Hence, more randomized controlled trials are needed to gain insight into the effectiveness of e-cigarettes for smoking cessation.

### Information Need on e-Cigarettes

In line with this ongoing debate, e-cigarette users, smokers, and nonusers reported that they have unanswered questions regarding e-cigarettes [16]. They raised questions about the harmfulness of e-cigarettes, especially compared to cigarette smoking, about the long-term health effects of e-cigarette use, and about e-cigarettes as a smoking cessation method. e-Cigarette users also report a lack of knowledge regarding the ingredients of e-cigarettes and its health effects [17]. Furthermore, incorrect risk perceptions regarding e-cigarette use and tobacco smoking are held by smokers. For instance, only half of the smokers believe that the use of e-cigarettes is less harmful than smoking tobacco [18], and fruit or candy flavors in e-cigarettes are perceived as less risky compared to tobacco flavors [19]. Thus, there is an information need regarding e-cigarettes, especially among smokers who may benefit from e-cigarettes as an aid in smoking cessation.

### Decision Making on e-Cigarettes

Owing to the uncertainty surrounding e-cigarettes, it is important that smokers have sufficient knowledge about e-cigarettes when deciding whether to use them. An informed choice is often defined based on relevant knowledge and the congruence between attitudes and conducted behavior [20]. These conceptualizations of informed decision making employ cut-off points in order to dichotomize constructs into positive and negative outcomes (eg, sufficient knowledge or not). These cut-off points are chosen arbitrarily, indicating that there is neither evidence for the choice of these cut-off points nor evidence that there is an underlying dichotomy at all [21]. Furthermore, individuals who score values close to the cut-off points but on opposite sites (eg, on a scale from 1-10, if 5 is considered to be the cut-off point, individuals who score values close to the cut-off point but on opposite sites would then for instance score 4.9 and 5.1) are categorized as being very different, while in reality being quite similar [21]. In this research, we will avoid dichotomizing continuous variables by examining the constructs of decision making separately.

### Research Goal

The goal of this study will be to assess the effect of tailored communication about e-cigarettes in a digital smoking cessation intervention on determinants of decision making, smoking reduction, and smoking cessation. In the context of a tailored eHealth program, smokers will be randomized into 1 of the 2 conditions—either receiving detailed tailored information about e-cigarettes or not. Information provision about e-cigarettes can have differential effects on smoking behavior, including favorable effects (eg, decreased number of tobacco cigarettes smoked, increased number of tobacco-abstinent participants) as well as unfavorable effects (eg, decreased number of tobacco-abstinent participants). Differences between conditions in the number of dual users (ie, people using e-cigarettes and smoking tobacco cigarettes) will be examined as well. Regarding decision making, we hypothesize that participants in the intervention condition will have more knowledge about e-cigarettes directly after the intervention compared to participants in the control condition. We did not formulate a hypothesis for the determinant attitude as neither a more positive nor a more negative attitude is directly associated with improved decision making. Regarding smoking behavior, we hypothesize that participants in the intervention condition will have smoked less tobacco cigarettes (adjusted for baseline measurement) in the past 7 days at the 6-month follow-up compared to participants in the control condition.

## Methods

### Study Design

**Figure 1:**
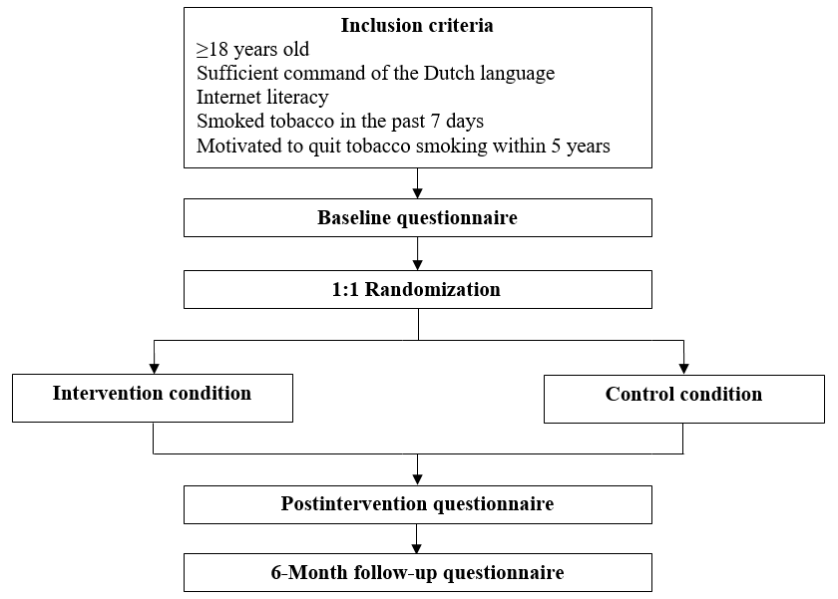
Flowchart of the study design.

A randomized controlled trial will be conducted and the results will be reported according to the CONSORT-EHEALTH checklist [22]. Participants will be 1:1 randomized into either the intervention condition or the control condition. Participants in both conditions will receive the same underlying digital smoking cessation intervention. The 2 conditions differ in the provision of information about e-cigarettes. Smokers in the intervention condition will receive detailed tailored information on e-cigarettes whereas smokers in the control condition will not receive that information. Measurements will be conducted at 3 points in time. A baseline questionnaire will be conducted at the start of the intervention. A first follow-up questionnaire will be conducted directly after completion of the intervention (ie, postintervention). A second follow-up questionnaire will be conducted at 6 months from the baseline. All questionnaires will be web-based and self-assessed. Figure 1 shows the study design.

### Participants and Recruitment

Inclusion criteria were that participants are at least 18 years old, have sufficient command of the Dutch language, have necessary internet literacy to use the intervention, have smoked tobacco in the past 7 days, and are motivated to quit tobacco smoking within 5 years. Participants were recruited using multiple strategies. A Dutch research agency was consulted in order to recruit smokers from their participant pool. Google Ads were used to recruit people who were searching the Google search engine for terms around smoking cessation. Social media and smoking-related forums were approached to recruit members of those channels. Moreover, flyers were distributed door-to-door in the Maastricht region, the Netherlands. Incentives were provided to participants who took part in the intervention and who answered all the questionnaires (baseline, postintervention, 6-month follow-up). Ten gift vouchers of €25 (US $1=€0.83) were raffled off among all participants who were recruited organically. Participants stemming from the research agency collected points within the system of the research agency, which could be exchanged for gift vouchers or donations. Interested individuals were directed to an external intervention website. Potential participants were informed that they would receive tailored smoking cessation advice during the intervention. The nature of tailoring was explained to clarify that the advice will be based on the answers participants provide to the questions during the intervention. The aim of this study was stated as exploring the opinion of smokers on the intervention. e-Cigarettes were not mentioned in the participant information text. Potential participants were informed about the possibility to withdraw from the study at any time without providing any reason. Participants did not need to register on the intervention website in order to limit the participation burden. After giving web-based informed consent, the inclusion criteria were verified by a short questionnaire. The intervention would take about 20 minutes (including the baseline and postintervention questionnaire). Answering the 6-month follow-up questionnaire will take about 3 minutes.

### Sample Size Calculation

The sample size was calculated using the ufs package [23] in R. Acknowledging that the accurate estimation of effect sizes is more important than relying on *P* values, we based our sample size calculation on accuracy in parameter estimation for Cohen *d* [24]. Unfortunately, we cannot infer the effect size from earlier research since we are not aware of any prior studies assessing the influence of providing information about e-cigarettes in a digital intervention on decision making and smoking cessation. Thus, we assumed a small effect size as it is usually found in digital health research on smoking cessation interventions [25]. Taking into account the small effect size of Cohen *d* of 0.2, a margin of error (half-width) of 0.15, and a confidence level of 95%, a total sample size of 687 participants is required.

### Intervention

**Figure 2:**
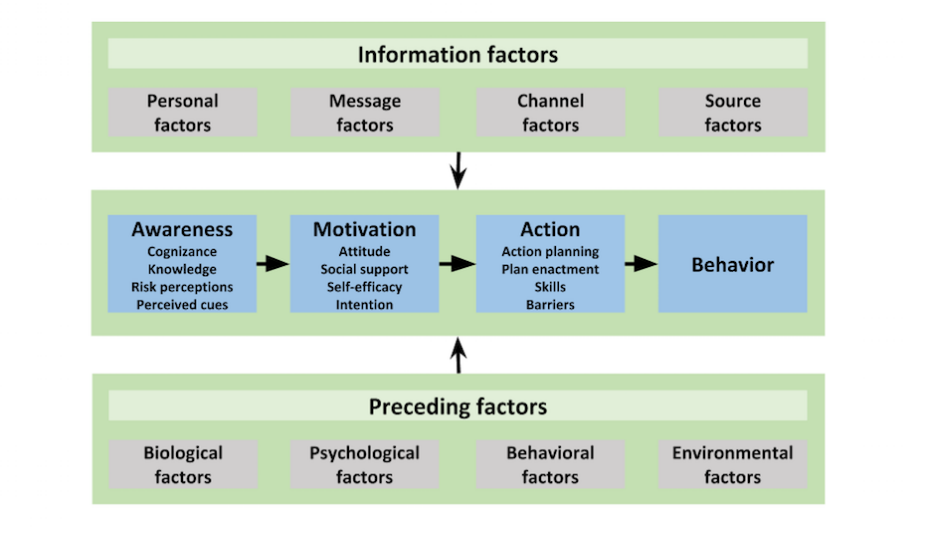
I-Change model [34].

The intervention will be a digital computer-tailored smoking cessation intervention that will be partly based on an earlier developed intervention at Maastricht University [26-28]. Compared to generic information, computer-tailored interventions provide highly individualized information that is tailored to the motivational and behavioral characteristics of the recipient [29]. According to the elaboration likelihood model, information that is perceived as personally relevant is expected to lead to more in-depth processing and, in turn, to more sustained attitudinal and behavioral changes [30]. The computer-tailored intervention will be based on the I-Change model [31,32], a comprehensive model that integrates various social-cognitive theories (see Figure 2). During the intervention, participants in both conditions will receive tailored advice on the pros and cons of quitting smoking (ie, attitude), social influence, preparatory plans, self-efficacy, and coping plans concerning smoking cessation. Participants will be able to decide based on their own interests and needs on which determinants of smoking cessation they would like to receive tailored advice. The information for the tailoring process is gathered by means of questionnaires that the recipient has to fill in during the intervention. Subsequently, a computerized process, employing if-then rules, selects appropriate feedback messages from a pool of all messages based on the answers that the recipient has given in the questionnaires [29,33].

**Figure 3:**
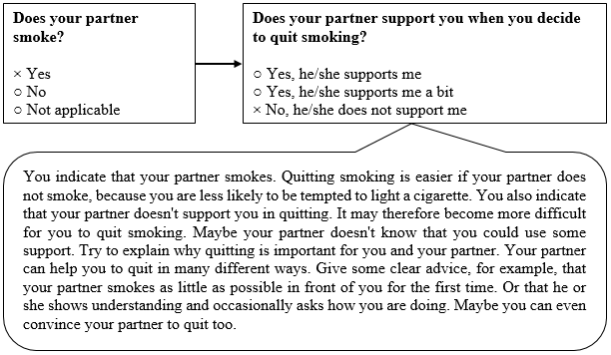
An example of tailored advice about the social influence of the partner.

The items of the questionnaires are based on previous research [27,28] (Elling and de Vries, unpublished data, 2021) and are reported in Multimedia Appendix 1. The pros and cons of quitting smoking (eg, “If I stop smoking, my physical fitness will improve”) will be assessed by 16 items. Social influence will consist of 2 components with 2 items each: social modeling (eg, “Does your partner smoke?”) and social support (eg, “Does your partner support you when you decide to quit smoking?”). Figure 3 illustrates an example of tailored advice for social support. Preparatory plans (eg, “I am planning to stop smoking completely without cutting down on cigarettes first”) will be assessed by 5 items. Self-efficacy (eg, “I find it difficult not to smoke if I am stressed”) will be assessed by 11 items. Coping plans (eg, “I have made clear plans to make sure that I will not smoke if I am stressed”) will be assessed by 11 items, reflecting the same situations as assessed for self-efficacy. After answering and receiving information on the determinants of smoking cessation, participants in both conditions will be able to indicate about which 6 clusters of smoking cessation methods they want to receive information (face-to-face counselling, eHealth interventions, telephone counselling, group-based programs, nicotine replacement therapy, and prescription medication).

**Figure 4:**
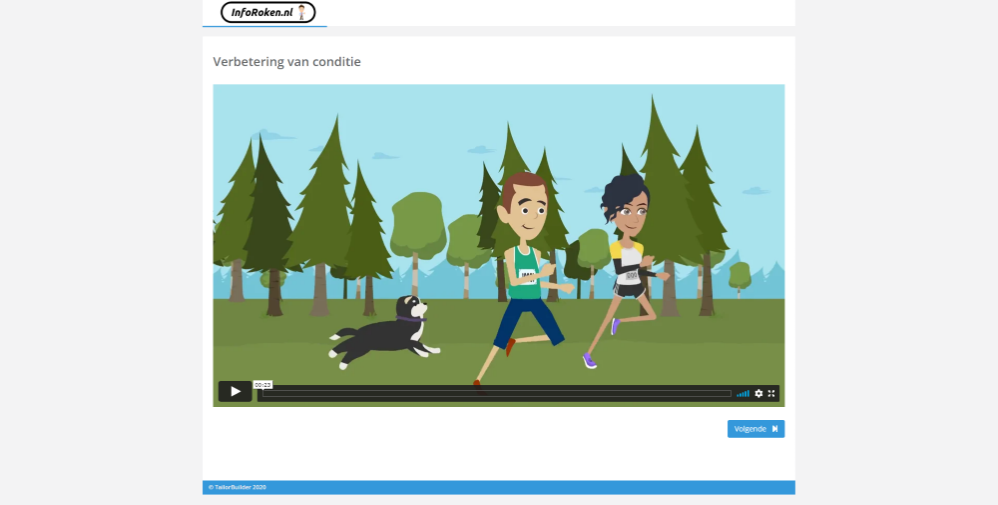
Screenshot of a webpage of the intervention showing an animated video advice.

**Figure 5:**
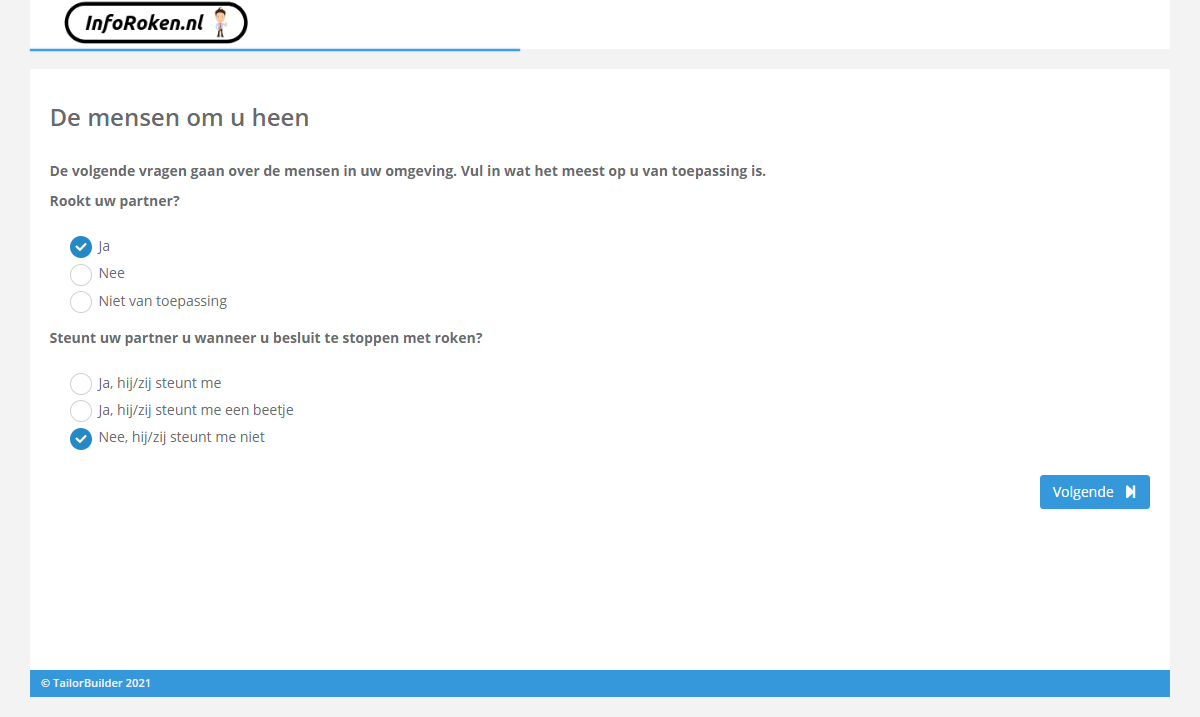
Screenshot of a webpage of the intervention showing 2 questions with answer options.

All advices concerning the pros and cons of quitting smoking, social influence, preparatory plans, self-efficacy, and coping plans will be presented in the form of spoken animations with little on-screen text in order to increase user experience and user engagement [34] (Elling and de Vries, unpublished data, 2021). A screenshot of an example of a webpage of the intervention with an animation is shown in Figure 4. All texts will be written in simple language and no hyperlinks to other resources will be presented. The website will be developed employing responsive web design and will thus be accessible on all common devices (eg, computer, smartphone) with all types of screen sizes. A second screenshot of a typical webpage presenting 2 questions of the tailoring process is shown in Figure 5.

### Tailored Information on e-Cigarettes

Participants in the intervention condition will receive tailored information on e-cigarettes based on 5 items (Do you know what an e-cigarette is? How harmful do you think e-cigarettes are compared to tobacco cigarettes? Do you think e-cigarettes are helpful in quitting smoking? Do you think using e-cigarettes is difficult or easy? Have you seen reports in the media about illnesses and deaths in the United States related to the use of e-cigarettes?). These items were developed by the research team and evaluated for comprehensibility and clarity by a communication expert of the National Institute for Public Health and the Environment. In general, the information will convey the message that, for smokers, the use of e-cigarettes is less harmful than continuing smoking tobacco cigarettes. However, it will be highlighted that this does not mean that using e-cigarettes is harmless. Regarding smoking cessation, it will be stressed that e-cigarettes are especially interesting for smokers who have tried to quit several times but have not succeeded. The possibility to (gradually) decrease the nicotine content of the e-cigarette liquid in order to cope with nicotine withdrawal symptoms will be discussed. The outbreak of lung injury associated with e-cigarette use in the United States of America will be discussed in detail. Participants in the control condition will receive a short text explaining that e-cigarettes are not actively recommended for smoking cessation (“A rather recent method that can be used to quit smoking is the e-cigarette. There is still a lot of uncertainty surrounding the e-cigarette. The e-cigarette is therefore not actively recommended as a method to quit smoking in the Netherlands.”). This short text is aimed to resemble the status quo of communication on e-cigarettes in smoking cessation interventions in the Netherlands.

### Measures

All items of the baseline questionnaire, postintervention questionnaire, and 6-month follow-up questionnaire are reported in Multimedia Appendix 2.

#### Smoking Reduction and Abstinence

The primary outcome of this study will be the number of tobacco cigarettes smoked in the past 7 days [35]. Secondary outcomes will be the average number of tobacco cigarettes smoked per day [36], 7-day point prevalence tobacco abstinence [37], and 7-day point prevalence e-cigarette abstinence [37]. If participants indicate to have used an e-cigarette, the nicotine content of the e-cigarette will be assessed. All outcomes will be assessed at baseline and at 6-month follow-up.

#### Smoking Cessation Methods

The *intention to use a smoking cessation method* (split-up per method) will be assessed directly after the intervention on a 5-point Likert scale ranging from 1=definitely do not to 5=definitely do. At the 6-month follow-up, we will assess which smoking cessation methods were actually utilized (ie, *smoking cessation method chosen*) on a dichotomous scale (yes/no). The following methods will be assessed: face-to-face counselling, eHealth interventions, telephone counselling, group-based programs, nicotine replacement therapy, prescription medication, and e-cigarettes. Participants can also indicate to have used another smoking cessation method or to not have used any smoking cessation method at all.

#### Determinants of Decision Making

Determinants of decision making will be assessed by 2 constructs: knowledge and attitude. Knowledge about e-cigarettes (eg, There are less harmful substances in e-cigarettes compared to tobacco cigarettes) will be measured by 7 items with response options being 1=True, 2=False, and 3=I do not know. Correct answers will be coded as 1 and incorrect answers and the option I do not know as 0. The sum of the correct answers is the overall score for the construct knowledge. Attitude on e-cigarettes (eg, I think that using e-cigarettes is better for my health than smoking cigarettes) will be measured by 10 items on a 5-point Likert scale ranging from 1=I totally disagree to 5=I totally agree. All items will be assessed directly after the intervention.

#### Process Evaluation

A process evaluation will be conducted by assessing an *overall grade* for the intervention [27], asking open questions about positive and negative aspects of the intervention, and by analyzing system usage data [27]. An *overall* g*rade* will be measured by 1 item on a scale ranging from 1=very bad to 10=very good. The open questions (eg, What do you like about the intervention?) will be asked to capture aspects that are perceived as both positively and negatively. The overall grade and the open questions will be assessed directly after the intervention. The time spent on the intervention website and the device (eg, smartphone, tablet, desktop) of the users will be measured using the TailorBuilder software (OverNite Software Europe BV). The time spent on the website will be provided per condition, whereas the device used will be reported for all participants together.

#### Demographics and Smoking Characteristics

We will assess the demographics by asking for *gender* (0=male, 1=female, 3=not on the list), *age*, and *education level* (1=low, 2=intermediate, 3=high). *Addiction level* will be assessed by the Fagerström Test for Nicotine Dependence [38]. The 6 items of the scale will be summed into an overall score ranging from 0 to 10. We will classify the dependence level as 0-2=low, 3-4=moderate, 5-6=strong, and 7-10=very strong. Addiction level will be measured at baseline. The *intention to quit smoking* will be assessed by 2 items. First, participants will be asked when they are planning to quit smoking (1=within 1 month, 2=within 6 months, 3=within 1 year, 4=within 5 years) [39]. Second, participants will be asked to indicate whether they are planning to quit smoking within 1 year on a 5-point Likert scale ranging from 1=definitely do not to 5=definitely do. The intention to quit smoking will be measured at baseline and after the intervention for every participant and at 6-month follow-up for participants who indicated that smoking cessation was not successful.

#### COVID-19 Pandemic and Smoking Behavior

The COVID-19 pandemic coincides with the recruitment and follow-up period of this research project. Participants are influenced by the pandemic in numerous ways, including the information that tobacco smoking may increase susceptibility to and severity of COVID-19 [40]. Thus, we included 15 items about smoking-related beliefs and behavior in times of COVID-19. These items are reported in Multimedia Appendix 3.

### Analyses

The focus of all the analyses will be on the effect size accompanied by the confidence interval [41]. Multiple imputations will be conducted to account for the missing observations at 6-month follow-ups. Sensitivity analyses will be conducted for complete cases and intention-to-treat [42]. The primary outcome (number of tobacco cigarettes smoked in past 7 days) will be tested by analysis of covariance [43,44]. The dependent variable will be the number of tobacco cigarettes smoked weekly at the 6-month follow-up. The number of tobacco cigarettes smoked weekly at baseline will be included as the covariate. The independent variable will be the condition. The average number of tobacco cigarettes smoked per day will be tested similarly. Logistic regression analyses will be performed to assess the effect of the intervention condition and control condition on 7-day point prevalence tobacco abstinence and 7-day point prevalence e-cigarette abstinence. Analyses of variance will be performed to test for differences in the determinants of decision making (knowledge and attitude on e-cigarettes) between conditions. Addiction level will be included as a covariate in additional sensitivity analyses. Previous research suggests that the addiction level needs to be considered when assessing the effectiveness of e-cigarettes for smoking reduction and cessation [45]. The open questions will be analyzed per question. Codes for recurrent themes will be created and reported in a table with example quotes and the number of times a theme was addressed.

## Results

The study is registered in the Netherlands Trial Register [46]. Ethical approval was granted by the Ethics Review Committee Health, Medicine and Life Sciences (FHML-REC) at Maastricht University (FHML-REC/2019/072). This project is supported by a research grant of the National Institute for Public Health and the Environment (Rijksinstituut voor Volksgezondheid en Milieu). Recruitment began in March 2020 and was completed by July 2020. We enrolled 492 smokers in this study. The results are expected to be published in June 2021.

## Discussion

Governmental public health institutes inform the public about smoking cessation. Usually, only information on the *best* option to quit smoking is provided, which is complete smoking cessation using evidence-based smoking cessation methods. Smokers may not follow this advice and they may do nothing about cessation, thereby making it the *worst* option. Smokers may also seek alternative advices for the *second best* option, which can be using e-cigarettes for smoking reduction and cessation. However, information about e-cigarettes is mostly not included in governmental smoking cessation interventions. Including information on e-cigarettes in smoking cessation interventions can yield different effects, which can be both favorable and detrimental to smokers specifically and public health in general. On the one hand, communication about e-cigarettes could lead to more people quitting smoking with the help of e-cigarettes, thereby reducing the number of people choosing the worst option. On the other hand, communication about e-cigarettes could lead to more people choosing the second best option who would otherwise have chosen the best option. This protocol describes a randomized controlled trial that aims to investigate the effects of including tailored information about e-cigarettes on decision making and smoking behavior. These findings can inform the development of future smoking cessation interventions, in particular, and communication about the second best option, in general.

## Appendix

Multimedia Appendix 1 Determinants of smoking cessation tackled in the intervention.

Multimedia Appendix 2 Items of the baseline, postintervention, and 6-month follow-up questionnaires.

Multimedia Appendix 3 Questionnaire items about smoking-related beliefs and behavior in times of COVID-19.

## Abbreviations

e-cigarette: electronic cigarette
