# Supplementary material for: Effects of Providing Tailored Information About e-Cigarettes in a Web-Based Smoking Cessation Intervention: Protocol for a Randomized Controlled Trial
Source: JMIR Res Protoc. 2021 May 14;10(5):e27088. doi: 10.2196/27088 (PMC8164120; doi:10.2196/27088)
Supplement: Multimedia Appendix 1 [file resprot_v10i5e27088_app1.docx]

# Multimedia Appendix 1: Determinants of smoking cessation tackled in the intervention

| Attitude (Pros) | |
| --- | --- |
| 1. | Improvement of physical fitness |
| 2. | Being an example to others |
| 3. | Improvement of health |
| 4. | Feeling more attractive |
| 5. | Being proud |
| 6. | Health of others |
| 7. | Saving money |
| 8. | Causing less nuisance to others |
|  | |
| Attitude (Cons) | |
| 1. | Difficulties relaxing |
| 2. | Gaining weight |
| 3. | Being bored more often |
| 4. | Feeling gloomy |
| 5. | Feeling insecure |
| 6. | Feeling stressed |
| 7. | Being less sociable |
| 8. | Getting withdrawal symptoms |
|  |  |
| Social influence (Social modeling & social support) | |
| Does … smoke? & Does … support you if you decide to quit smoking? | |
| 1 | Smoking behavior of partner |
| 2 | Smoking behavior of people in the social environment |
|  | |
| Preparatory plans | |
| I am planning… | |
| 1 | …to stop completely without cutting down on cigarettes first |
| 2 | …to dispose all smoking related things from my house |
| 3 | …to ask my guests to not smoke in my presence |
| 4 | …to tell others that I will stop smoking |
|  | |
| Self-efficacy & Coping plans | |
| I find it difficult not to smoke… & I have made plans to make sure that I will not smoke… | |
| 1 | …if I am stressed |
| 2 | …if I am mad |
| 3 | …if I am sad |
| 4 | …if somebody offers me a cigarette |
| 5 | …if I see somebody enjoying a cigarette |
| 6 | …if I am at a party |
| 7 | …if I am drinking tea or coffee |
| 8 | …after I have eaten |
| 9 | …if I am having a break |
| 10 | …if I get up in the morning |
| 11 | …if I feel like needing a cigarette |
