# Supplementary material for: Effects of Providing Tailored Information About e-Cigarettes in a Web-Based Smoking Cessation Intervention: Protocol for a Randomized Controlled Trial
Source: JMIR Res Protoc. 2021 May 14;10(5):e27088. doi: 10.2196/27088 (PMC8164120; doi:10.2196/27088)
Supplement: Multimedia Appendix 2 [file resprot_v10i5e27088_app2.docx]

# Multimedia Appendix 2: Items of the baseline, post intervention, and 6-month follow-up questionnaires

| **Baseline Questionnaire** | | | | |
| --- | --- | --- | --- | --- |
| **Part** | **Title** | **Question** | **Answer options** | **Coding** |
| Informed Consent | Informed Consent | Do you want to participate? | [Yes, I want to participate and I give Maastricht University permission to use my data for scientific research]  [No, I do not give Maastricht University permission to use my data for scientific research and therefore do not participate in the study] | (1)  (2) |
| Inclusion criteria | Age | What is your age in years? | <age> |  |
|  | 7-day tobacco PPA | Have you smoked in the past 7 days? | [yes]  [no] | (1)  (2) |
|  | Motivation to quit | Do you want to quit smoking within 5 years? | [yes]  [no] | (1)  (2) |
|  | E-Mail | What is your e-mail address? We need it to send you an invitation for a second questionnaire after 6 months. | Free text input |  |
| Demographics | Gender | What is your gender? | [man]  [woman]  [not on the list] | (1)  (2)  (3) |
|  | Education level | What is your highest completed education? | [Primary education, vmbo, havo-onderbouw, wvo-onderbouw, mbo1]  [Havo, vwo, mbo]  [Hbo-, wo-bachelor/master] | (1)  (2)  (3) |
| Source | Source | How did you end up on this website? | [Google]  [Facebook]  [Flycatcher]  [Heard from family, friends, colleagues]  [Flyer]  [Anders] | (1)  (2)  (3)  (4)  (5)  (6) |
| Experience | Participated in intervention before | Have you participated in an online smoking cessation program before? | [yes]  [no] | (1)  (2) |
|  | *Pop-up when people tick "Yes”* | | | |
|  | Name program | Which program did you participate in? | Free text input |  |
| Addiction level | Fagerström 1 | How soon after you wake up do you smoke your first cigarette? | [Within 5 minutes]  [6 to 30 minutes]  [31 to 60 minutes]  [After 60 minutes] | (3)  (2)  (1)  (0) |
|  | Fagerström 2 | Do you find it difficult to refrain from smoking in places where it is forbidden (e.g., in  church, at the library, in the cinema)? | [yes]  [no] | (1)  (0) |
|  | Fagerström 3 | Which cigarette would you hate most to give up? | [The first one in the morning]  [Any other] | (1)  (0) |
|  | Fagerström 5 | Do you smoke more frequently during the first hours after waking than during the rest of the day? | [During the first hours]  [During the rest of the day] | (1)  (0) |
|  | Fagerström 6 | Do you smoke when you are so ill that you are in bed most of the day? | [yes]  [no] | (1)  (0) |
| Smoking behavior | Number of tobacco cigarettes smoked per day | How many regular cigarettes and/or roll-your-own cigarettes do you smoke on average per day? | <number> |  |
|  | Number of tobacco cigarettes smoked in the past 7 days | How many regular cigarettes and/or roll-your-own cigarettes have you smoked in the past 7 days? | <number> |  |
|  | 7-day e-cigarette PPA | Have you used an e-cigarette in the past 7 days? | [yes]  [no] | (1)  (2) |
|  | *Pop-up when people tick "Yes”* | | |  |
|  | Nicotine | Have you mainly used a liquid with or without nicotine? | [With nicotine]  [Without nicotine]  [I do not know] | (1)  (2)  (3) |
| Intention to quit smoking | TTM | When do you want to stop smoking? Choose the option that applies most to you. | [Within 1 month]  [Within 6 months]  [Within 1 year]  [Within 5 years] | (1)  (2)  (3)  (4) |
|  | TPB | Are you planning to quit smoking within 1 year? | [No, definitely not]  No, probably not]  [Maybe]  [Yes, probably]  [Yes, definitely] | (1)  (2)  (3)  (4)  (5) |
| Quit attempts | Quit attempts | Have you tried to quit smoking in the past year? | [yes]  [no] | (1)  (2) |

| **Post Intervention Questionnaire** | | | | |
| --- | --- | --- | --- | --- |
| **Part** | **Title** | **Question** | **Answer options** | **Coding** |
| Intention to quit smoking | TTM | You may have changed your mind about quitting smoking after using this program. That's why we ask you again when you want to stop smoking? Choose the option that applies most to you. | [Within 1 month]  [Within 6 months]  [Within 1 year]  [Within 5 years] | (1)  (2)  (3)  (4) |
|  | TPB | Are you planning to quit smoking within 1 year? | [No, definitely not]  No, probably not]  [Maybe]  [Yes, probably]  [Yes, definitely] | (1)  (2)  (3)  (4)  (5) |
|  | Intention to use specific smoking cessation method | Are you planning to use the following smoking cessation methods? | | |
|  |  | Face-to-face counselling | [Nee, zeker niet]  [Nee, niet]  [Misschien]  [Ja, wel]  [Ja, zeker wel] | (1)  (2)  (3)  (4)  (5) |
|  |  | eHealth intervention | [No, definitely not]  No, probably not]  [Maybe]  [Yes, probably]  [Yes, definitely] | (1)  (2)  (3)  (4)  (5) |
|  |  | Telephone counselling | [No, definitely not]  No, probably not]  [Maybe]  [Yes, probably]  [Yes, definitely] | (1)  (2)  (3)  (4)  (5) |
|  |  | Group-based program | [No, definitely not]  No, probably not]  [Maybe]  [Yes, probably]  [Yes, definitely] | (1)  (2)  (3)  (4)  (5) |
|  |  | E-cigarette (mainly with nicotine) | [No, definitely not]  No, probably not]  [Maybe]  [Yes, probably]  [Yes, definitely] | (1)  (2)  (3)  (4)  (5) |
|  |  | E-cigarette (mainly without nicotine) | [No, definitely not]  No, probably not]  [Maybe]  [Yes, probably]  [Yes, definitely] | (1)  (2)  (3)  (4)  (5) |
|  |  | Nicotine replacement therapy | [No, definitely not]  No, probably not]  [Maybe]  [Yes, probably]  [Yes, definitely] | (1)  (2)  (3)  (4)  (5) |
|  |  | Prescription medication | [No, definitely not]  No, probably not]  [Maybe]  [Yes, probably]  [Yes, definitely] | (1)  (2)  (3)  (4)  (5) |
|  |  | Another cessation method, namely... | | |
| Knowledge | Knowledge regarding e-cigarettes | In your opinion, are the following statements false or true? | | |
|  |  | E-cigarettes contain tobacco (R) | [False]  [I do not know]  [True] | (1)  (2)  (3) |
|  |  | E-cigarettes contain less harmful substances than regular cigarettes | [False]  [I do not know]  [True] | (1)  (2)  (3) |
|  |  | E-cigarettes with nicotine are addictive | [False]  [I do not know]  [True] | (1)  (2)  (3) |
|  |  | The vapor of e-cigarettes consists only of water (R) | [False]  [I do not know]  [True] | (1)  (2)  (3) |
|  |  | The use of e-cigarettes can cause irritation and damage to the respiratory tract | [False]  [I do not know]  [True] | (1)  (2)  (3) |
|  |  | For smokers, the use of e-cigarettes is less harmful than continuing to smoke | [False]  [I do not know]  [True] | (1)  (2)  (3) |
|  |  | The long-term effects of the use of e-cigarettes have not been sufficiently researched | [False]  [I do not know]  [True] | (1)  (2)  (3) |
| Attitude | Attitude toward substituting e-cigarettes for cigarettes | To what extent do you agree with the following statements? | | |
|  |  | I think that it is wise to use e-cigarettes instead of smoking regular cigarettes | [Strongly disagree]  [Disagree]  [Neither agree nor disagree]  [Agree]  [Strongly agree] | (1)  (2)  (3)  (4)  (5) |
|  |  | I think that the use of e-cigarettes is better for my health than smoking regular cigarettes | [Strongly disagree]  [Disagree]  [Neither agree nor disagree]  [Agree]  [Strongly agree] | (1)  (2)  (3)  (4)  (5) |
|  |  | I think that the use of e-cigarettes is better for the health of the people around me than smoking regular cigarettes | [Strongly disagree]  [Disagree]  [Neither agree nor disagree]  [Agree]  [Strongly agree] | (1)  (2)  (3)  (4)  (5) |
|  | Attitude toward e-cigarettes for smoking reduction and cessation | To what extent do you agree with the following statements? | | |
|  |  | I think that e-cigarettes can help me to smoke less | [Strongly disagree]  [Disagree]  [Neither agree nor disagree]  [Agree]  [Strongly agree] | (1)  (2)  (3)  (4)  (5) |
|  |  | I think that e-cigarettes can help me to quit smoking | [Strongly disagree]  [Disagree]  [Neither agree nor disagree]  [Agree]  [Strongly agree] | (1)  (2)  (3)  (4)  (5) |
|  |  | I think that the use of e-cigarettes increases my chances of successfully quitting smoking | [Strongly disagree]  [Disagree]  [Neither agree nor disagree]  [Agree]  [Strongly agree] | (1)  (2)  (3)  (4)  (5) |
|  | Attitude toward e-cigarettes in general | To what extent do you agree with the following statements? | | |
|  |  | I think that e-cigarettes can help me to quench my cravings for regular cigarettes | [Strongly disagree]  [Disagree]  [Neither agree nor disagree]  [Agree]  [Strongly agree] | (1)  (2)  (3)  (4)  (5) |
|  |  | I think that e-cigarettes taste good | [Strongly disagree]  [Disagree]  [Neither agree nor disagree]  [Agree]  [Strongly agree] | (1)  (2)  (3)  (4)  (5) |
|  |  | I think that e-cigarettes are easy to use | [Strongly disagree]  [Disagree]  [Neither agree nor disagree]  [Agree]  [Strongly agree] | (1)  (2)  (3)  (4)  (5) |
|  |  | I think that I smell less like smoke when I use e-cigarettes instead of regular cigarettes | [Strongly disagree]  [Disagree]  [Neither agree nor disagree]  [Agree]  [Strongly agree] | (1)  (2)  (3)  (4)  (5) |
| Process evaluation | Overall grade | What is the overall grade you would give to the online program? (where 10 is the best) | <number> | 1-10 |
|  | Positive aspects | What did you like about the online program? | Free text input |  |
|  | Negative aspects | What did you dislike about the online program? | Free text input |  |

| **6-Month Follow-up Questionnaire** | | | | |
| --- | --- | --- | --- | --- |
| **Part** | **Title** | **Question** | **Answer options** | **Coding** |
| Smoking cessation | Quit attempt | Since you participated in the study (6 months ago), have you seriously tried to quit smoking (at least 24 hours without smoking a regular cigarette and/or roll-your-own cigarette)? | [yes]  [no] | (1)  (2) |
|  | *Pop-up if participants tick "Yes”* | | | |
|  | Number of quit attempts | How many times have you seriously tried to quit smoking since you took part in the study (at least 24 hours without smoking a regular cigarette and/or roll-your-own cigarette)? | <number> |  |
|  | 24-hour tobacco PPA | Have you smoked one or more regular cigarettes and/or roll-your-own cigarettes in the last 24 hours? | [yes]  [no] | (1)  (2) |
|  | 7-day tobacco PPA | Have you smoked one or more regular cigarettes and/or roll-your-own cigarettes in the past 7 days? | [yes]  [no] | (1)  (2) |
|  | *Pop-up question if participants tick "Yes", otherwise automatically #cigarettes = 0* | | | |
|  | Average number of tobacco cigarettes smoked per day | How many regular cigarettes and/or roll-your-own cigarettes have you smoked on average per day? | <number> |  |
|  | Number of tobacco cigarettes smoked in the past 7 days | How many regular cigarettes and/or roll-your-own cigarettes have you smoked in the past 7 days? | <number> |  |
|  | *If participants still smoke (7-day tobacco PPA) but have tried to quit (quit attempt)* | | | |
|  | Time to first relapse | How many days after you seriously tried to quit smoking for the first time have you smoked on 7 consecutive days? | <number> |  |
|  | 7-day e-cigarette PPA | Have you used an e-cigarette in the past 7 days? | [yes]  [no] | (1)  (2) |
|  | *If participants have used an e-cigarette* | | | |
|  | Frequency | How often have you used an e-cigarette in the past 7 days? | [Every day]  [Several times]  [Once] | (1)  (2)  (3) |
|  | Nicotine | Have you mainly used a liquid with or without nicotine? | [With nicotine]  [Without nicotine]  [I do not know] | (1)  (2)  (3) |
| Intention to quit smoking | *Only when people are still smoking (7-day tobacco PPA)* | | | |
|  | TTM | When do you want to quit smoking? Choose the option that applies most to you. | [Within 1 month]  [Within 6 months]  [Within 1 year]  [Within 5 years]  [I don't want to quit smoking anymore] | (1)  (2)  (3)  (4)  (5) |
|  | TPB | Are you planning to quit smoking within 1 year? | [No, definitely not]  No, probably not]  [Maybe]  [Yes, probably]  [Yes, definitely] | (1)  (2)  (3)  (4)  (5) |
| Smoking cessation methods chosen | Smoking cessation methods chosen | Which smoking cessation methods did you use? | | |
|  |  | Face-to-face counselling | [yes]  [no] | (1)  (2) |
|  |  | eHealth intervention | [yes]  [no] | (1)  (2) |
|  |  | Telephone counselling | [yes]  [no] | (1)  (2) |
|  |  | Group-based program | [yes]  [no] | (1)  (2) |
|  |  | E-cigarette | [yes]  [no] | (1)  (2) |
|  |  | Nicotine replacement therapy | [yes]  [no] | (1)  (2) |
|  |  | Prescription medication | [yes]  [no] | (1)  (2) |
|  |  | Another smoking cessation method, namely… | Free text input |  |
| E-cigarette | *Only if people have used an e-cigarette* | | | |
|  | Nicotine | Have you mainly used a liquid with or without nicotine? | [With nicotine]  [Without nicotine]  [I do not know] | (1)  (2)  (3) |
|  |  | To what extent do you agree with the following statement?  I have tried to reduce my nicotine addiction by using less and less nicotine in the liquid. | [Strongly disagree]  [Disagree]  [Neither agree nor disagree]  [Agree]  [Strongly agree] | (1)  (2)  (3)  (4)  (5) |
|  | Experience | To what extent do you agree with the following statements? | | |
|  |  | The e-cigarette helped me to quit smoking | [Strongly disagree]  [Disagree]  [Neither agree nor disagree]  [Agree]  [Strongly agree] | (1)  (2)  (3)  (4)  (5) |
|  |  | I would recommend the e-cigarette to a friend who wants to quit smoking | [Strongly disagree]  [Disagree]  [Neither agree nor disagree]  [Agree]  [Strongly agree] | (1)  (2)  (3)  (4)  (5) |
|  |  | What did you like about the e-cigarette? | Free text input |  |
|  |  | What did you dislike about the e-cigarette? | Free text input |  |
