# Supplementary material for: Effects of Providing Tailored Information About e-Cigarettes in a Web-Based Smoking Cessation Intervention: Protocol for a Randomized Controlled Trial
Source: JMIR Res Protoc. 2021 May 14;10(5):e27088. doi: 10.2196/27088 (PMC8164120; doi:10.2196/27088)
Supplement: Multimedia Appendix 3 [file resprot_v10i5e27088_app3.docx]

# Multimedia Appendix 3: Questionnaire items about smoking related beliefs and behavior in times of COVID-19

Items 1-11 will be measured at baseline. Items 1-10 will be measured on a 5-point Likert scale ranging from 1=‘Strongly disagree’ to 5=‘Strongly agree’. Item 11 will be measured with response options being 1=‘Less cigarettes’, 2=‘Unchanged’, and 3=‘More cigarettes’. For response options 1 and 3, the exact number of cigarettes smoked less or more will be measured as well.

Items 12-15 will be measured at 6-month follow-up. Item 12, 13, 15 will be measured on a 5-point Likert scale ranging from 1=‘Strongly disagree’ to 5=‘Strongly agree’. Item 14 will be measured with response options being 1=‘Less cigarettes’, 2=‘Unchanged’, and 3=‘More cigarettes’.

1. The coronavirus is a serious threat to my health
2. Compared to non-smokers, I have a higher risk of getting severe complaints from the coronavirus
3. The chances that I will get the coronavirus are high
4. Compared to non-smokers, the chances are higher that I will get the coronavirus
5. If I stop smoking, I reduce the chances of serious complaints due to the coronavirus
6. My environment thinks that I should quit smoking because of the coronavirus
7. Since the coronavirus outbreak I experience more stress in my daily life
8. Because of the coronavirus, I find it hard to quit smoking
9. Because of the coronavirus, I'm now more motivated to make plans to quit smoking
10. Because of the coronavirus, I'm now more motivated to quit smoking
11. Because of the coronavirus, I now smoke more/less a day
12. I stopped smoking (mainly) because of the coronavirus
13. I had stopped smoking but I started again (mainly) because of the coronavirus
14. Because of the coronavirus, I now smoke more/less a day
15. I started using an e-cigarette because of the coronavirus
